# Serum Vitamin D Levels at Birth and Late-Onset Neonatal Sepsis in Preterm Neonates: A Retrospective Exploratory Cohort Study

**DOI:** 10.3390/children13060727

**Published:** 2026-05-23

**Authors:** Esteban López-Garrido, Alejandra Luna-Huerta, Ana Patricia Ortega-González, Hadassa Yuef Martínez-Padrón

**Affiliations:** 1 Unidad de Cuidados Intensivos en Neonatos, Hospital Regional de Alta Especialidad Ciudad Victoria, Servicios de Salud del Instituto Mexicano del Seguro Social Para el Bienestar (IMSS-BIENESTAR), Libramiento Guadalupe Victoria S/N, Área de Pajaritos, Victoria 87087, Tamaulipas, Mexico; estebanlopez_garrido@hotmail.com; 2 Facultad de Medicina, Universidad Autónoma de Tamaulipas, Matamoros 87300, Tamaulipas, Mexico or a2193620144@alumnos.uat.edu.mx (A.L.-H.); or a2193620145@alumnos.uat.edu.mx (A.P.O.-G.); 3 Subdirección de Enseñanza e Investigación, Hospital Regional de Alta Especialidad Ciudad Victoria, Servicios de Salud del Instituto Mexicano del Seguro Social Para el Bienestar (IMSS-BIENESTAR), Libramiento Guadalupe Victoria S/N, Área de Pajaritos, Victoria 87087, Tamaulipas, Mexico

**Keywords:** vitamin D, 25-hydroxyvitamin D, preterm neonates, late-onset neonatal sepsis, neonatal intensive care unit, prematurity

## Abstract

**Highlights:**

**What are the main findings?**

- Serum 25-hydroxyvitamin D levels at birth were predominantly sufficient in preterm neonates.
- No significant association was found between vitamin D status at birth and late-onset neonatal sepsis.

**What are the implications of the main findings?**

- Vitamin D status at birth showed no clear association with late-onset neonatal sepsis in this exploratory cohort.
- Population-specific factors and methodological differences should be considered when interpreting the relationship between vitamin D and neonatal infectious outcomes.

**Abstract:**

**Background**: Late-onset neonatal sepsis (LONS) remains a major cause of morbidity in preterm neonates admitted to the neonatal intensive care unit (NICU), yet the contribution of vitamin D status to neonatal infectious susceptibility remains uncertain. **Objective**: To evaluate clinical and demographic variables and serum vitamin D levels assessed at birth in preterm neonates with and without LONS. **Methods**: A retrospective observational cohort study was conducted in a tertiary NICU in northeastern Mexico between May 2023 and October 2024. Preterm neonates (<37 weeks of gestation) with serum 25(OH)D measured within the first hour of life were included. Vitamin D status was classified as sufficient (≥30 ng/mL), insufficient (20–29 ng/mL), or deficient (<20 ng/mL). LONS was defined as sepsis occurring after 72 h of life. Comparisons between neonates with and without LONS were performed using Fisher’s exact test for categorical variables and Student’s *t*-test or Mann–Whitney U test for continuous variables, as appropriate. **Results**: Twenty-nine preterm neonates were included (mean gestational age: 32.0 ± 2.6 weeks; mean birth weight: 1748 ± 545 g). The mean serum 25(OH)D level at birth was 35.5 ± 13.0 ng/mL. LONS occurred in 31% (9/29) of neonates, of which 55% were microbiologically confirmed. No significant differences were observed in vitamin D levels between neonates with and without LONS (35.0 ± 12.0 vs. 35.7 ± 13.7 ng/mL; *p* = 0.899). Vitamin D deficiency was not associated with LONS (OR 1.13, 95% CI 0.09–14.28). The prevalence of vitamin D deficiency was low (10%) in this cohort. **Conclusions**: A clear association between serum 25(OH)D levels at birth and the development of LONS could not be demonstrated in this small exploratory cohort. Given the limited sample size and low prevalence of vitamin D deficiency, further multicenter prospective studies are required to better understand the potential relationship between vitamin D status and neonatal infectious outcomes.

## 1. Introduction

Neonatal sepsis (NS) remains a major cause of morbidity and mortality worldwide, particularly among preterm neonates. Globally, it affects approximately 4–22 newborns per 1000 live births, with an estimated three million cases each year [1]. Mortality rates range from 11% to 19% and may reach up to 24% in low-income countries [2]. Survivors are at increased risk of long-term neurodevelopmental complications, including cerebral palsy, hearing impairment, visual deficits, and cognitive delay [3]. NS is typically classified according to the time of onset as early-onset sepsis (EOS) or late-onset sepsis (LONS) [4]. LONS occurs after 72 h of life and is frequently associated with nosocomial acquisition in the NICU [5,6,7,8]. Preterm neonates represent the population at greatest risk due to the immaturity of their immune system and the frequent need for invasive procedures such as central venous catheters, endotracheal tubes, and prolonged parenteral nutrition [5,8,9,10]. The incidence of LONS may reach up to 20% among very low-birth-weight infants (<1500 g) and even higher among extremely low-birth-weight neonates [11,12].

Vitamin D has gained increasing attention in recent years due to its immunomodulatory properties [13,14,15]. Beyond its classical role in calcium and phosphorus metabolism, vitamin D participates in innate and adaptive immune responses, including modulation of T-lymphocyte activity and regulation of antimicrobial peptides such as cathelicidin [10,15,16,17,18]. Serum 25-hydroxyvitamin D [25(OH)D] is considered the most reliable marker of vitamin D status [19]. According to international guidelines, vitamin D levels are classified as deficient (<20 ng/mL), insufficient (20–29 ng/mL), or sufficient (≥30 ng/mL) [12,20].

Clinical sepsis is defined as the presence of two or more of the following signs: tachypnea, apnea, tachycardia, bradycardia, mottled skin, temperature instability, lethargy, irritability, vomiting, poor feeding, elevated C-reactive protein > 10 mg/L, serum procalcitonin > 2.5 ng/mL, leukocytosis > 25,000 or <5000, or thrombocytopenia (platelet count < 100 × 10^3^/µL) after 72 h. Confirmed sepsis is defined as clinical sepsis with a positive blood culture. Vitamin D deficiency is highly prevalent worldwide, including during pregnancy, and maternal vitamin D status directly influences fetal and neonatal levels [21,22,23]. Low vitamin D concentrations during pregnancy have been associated with adverse perinatal outcomes such as preeclampsia, preterm birth, respiratory morbidity, and low birth weight [23]. In neonates, vitamin D deficiency has also been linked to increased susceptibility to respiratory infections and immune-mediated conditions [23,24,25]. Several observational studies and meta-analyses have suggested a potential association between low neonatal or maternal vitamin D levels and an increased risk of NS [26,27]. However, the available evidence remains limited, heterogeneous, and controversial, and most studies have focused on term infants or EOS [28].

Preterm neonates admitted to a tertiary NICU represent a highly vulnerable and inherently limited population, in whom clinical research is frequently constrained by strict eligibility criteria, physiological instability, and ethical considerations [29,30,31,32,33]. In this context, studies are essential to generate preliminary evidence and identify clinically relevant associations that may inform subsequent hypothesis-driven research. Evidence linking vitamin D status at birth with LONS in preterm neonates remains limited and inconsistent, particularly in low- and middle-income countries such as Mexico, where regional data are scarce. Moreover, there is currently no evidence from Mexico assessing the potential association between neonatal vitamin D levels and LONS in preterm populations. Considering the potential immunological role of vitamin D and the vulnerability of premature infants to infectious complications, further research is needed to clarify this relationship. Therefore, the aim of this study was to evaluate clinical and demographic variables and serum vitamin D levels assessed at birth in preterm neonates with and without LONS.

## 2. Materials and Methods

### 2.1. Study Design and Procedures

This retrospective observational cohort study was based on the review of clinical records and laboratory data obtained during routine neonatal care in the NICU. Serum 25-hydroxyvitamin D [25(OH)D] measurements were not performed as part of a universal screening protocol for all preterm neonates admitted during the study period. Instead, testing was selectively requested as part of the institutional clinical evaluation according to clinical judgment and test availability. Consequently, only preterm neonates with available serum 25(OH)D measurements obtained within the first hour of life were eligible for inclusion in the present analysis. No additional blood samples were collected specifically for research purposes.

Due to the retrospective nature of the study, complete information regarding the total number of preterm births during the study period and the specific reasons why some neonates did not undergo vitamin D testing was not consistently available in the medical records. Therefore, the possibility of selection bias cannot be excluded.

**Figure 1:**
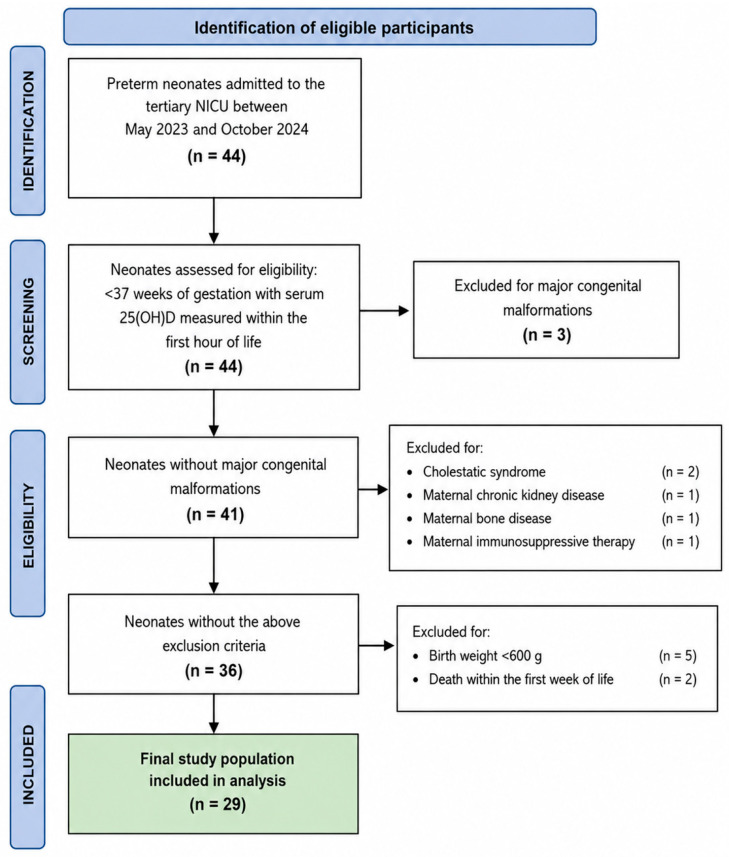
Flow diagram of patient selection, inclusion, and final study population.

Exclusion criteria included major congenital malformations, cholestatic syndrome, maternal chronic kidney disease, maternal bone disease, and maternal immunosuppressive therapy. Neonates weighing <600 g and those who died within the first week of life were also excluded because their extreme clinical instability and high early mortality limited the evaluation of LONS, which was defined as occurring after 72 h of life (Figure 1).

Vitamin D supplementation was administered according to the institutional neonatal nutritional protocol after achievement of full enteral feeding. Supplementation was generally initiated at a dose of 400 IU/day and subsequently adjusted based on individual nutritional requirements, enteral tolerance, growth parameters, and clinical condition. Because of the retrospective nature of the study, detailed longitudinal information regarding the exact timing of supplementation initiation, cumulative dose, duration of treatment, adherence, and serial monitoring of vitamin D levels was not consistently available in all patients. A blood sample of approximately 1 mL was collected within the first hour of life using a red microtainer tube (1.3 mL capacity), protected from light with aluminum foil. Blood sampling was performed either by umbilical arterial sampling performed immediately after birth using a 3 mL syringe with a 22-gauge needle or through an umbilical arterial catheter when available for clinical management, simultaneously with routine laboratory sampling. Samples were transported to the hospital clinical laboratory (LISTER) within five minutes of collection.

Serum 25(OH)D concentrations were measured using a chemiluminescence assay on a VITROS^®^ 3600 analyzer, following electronic test request through the hospital laboratory information system. Results are reported in ng/mL. Vitamin D status was classified as sufficient (≥30 ng/mL), insufficient (20–29 ng/mL), or deficient (<20 ng/mL) according to widely accepted international guidelines [12,20]. After analysis, any remaining biological material was discarded in accordance with the institutional protocol for the management of infectious biological waste established by the clinical laboratory service.

Clinical and demographic variables were recorded, including gestational age, birth weight, length, sex, Apgar score at birth, maternal morbidities (preeclampsia, diabetes, and chorioamnionitis), and neonatal morbidities such as LONS, respiratory distress syndrome, transient tachypnea of the newborn, pulmonary adaptation syndrome, and neonatal pneumonia. Nosocomial pneumonia was analyzed as a separate outcome. LONS was defined as a systemic infectious syndrome occurring after 72 h of life, characterized by clinical and laboratory evidence of infection requiring antimicrobial treatment. Clinical sepsis was defined as the presence of at least two compatible clinical signs suggestive of systemic infection occurring after 72 h of life, together with at least one abnormal laboratory parameter indicative of inflammation or infection, in the absence of microbiological confirmation. Clinical criteria included respiratory instability, apnea, bradycardia, tachycardia, temperature instability, feeding intolerance, lethargy, irritability, hemodynamic instability, or mottled skin. Laboratory abnormalities included elevated C-reactive protein (>10 mg/L), procalcitonin (> 2.5 ng/mL), leukocytosis (>25,000cells/mm^3^), leukopenia (<5000 cells/mm^3^), or thrombocytopenia (platelet count < 100 × 10^3^/µL).

Microbiologically confirmed sepsis was defined as the presence of clinical manifestations consistent with systemic infection together with microbiological evidence of infection, including a positive blood culture and/or cerebrospinal fluid culture. Although PCR-positive/culture-negative cases had been considered within the predefined criteria for microbiological confirmation, no such cases were identified in the final study cohort. Antibiotic therapy was initiated according to institutional neonatal sepsis management protocols based on clinical evaluation by the attending neonatologist. Neonates classified as clinical sepsis received antimicrobial treatment for suspected infection despite negative microbiological cultures.

All laboratory analyses were performed by trained personnel blinded to clinical outcomes. Finally, the association between vitamin D status and neonatal morbidity was analyzed.

### 2.2. Ethical Approval and Data Collection Procedures

This study was conducted in accordance with the Declaration of Helsinki and national regulations for research involving human subjects. The study consisted of a retrospective review of clinical records and laboratory data obtained during routine neonatal care in the neonatal intensive care unit (NICU) of the HRAEV between May 2023 and October 2024.

Serum 25-hydroxyvitamin D [25(OH)D] measurements were performed as part of routine institutional clinical evaluation. No additional blood samples or experimental procedures were performed for research purposes.

During part of the study period, the institutional Research and Ethics Committees of the HRAEV were undergoing administrative restructuring and re-registration processes under COFEPRIS regulations. Institutional authorization for retrospective review and academic analysis of previously collected clinical and laboratory information was granted by the HRAEV Research Committee under approval PT-007-2024 (25 September 2024). Additional academic and methodological oversight was provided by the Universidad Autónoma de Tamaulipas under approval POST-25-017 PED (22 January 2025). Institutional ethical ratification following committee restructuring was subsequently granted by the HRAEV Ethics Committee under approval PI-CEI-003-2026 (24 February 2026). All data were anonymized prior to analysis, and patient confidentiality was maintained throughout the study.

### 2.3. Statistical Analysis

Descriptive statistics were used to summarize the data. Continuous variables with a normal distribution are expressed as mean ± standard deviation (SD), while non-normally distributed variables are reported as median and interquartile range (IQR). Categorical variables are presented as frequencies and percentages.

Comparisons between neonates with and without LONS were performed using Student’s *t*-test for independent samples or the Mann–Whitney U test, as appropriate. Given the small sample size and low expected cell counts, Fisher’s exact test was used for categorical variables.

Unadjusted odds ratios (ORs) with corresponding 95% confidence intervals (CIs) were calculated using 2 × 2 contingency tables to explore the association between vitamin D deficiency and LONS. Multivariable adjustment was not performed due to the exploratory nature of the study and the limited sample size. Given the small sample size and sparse cell counts, OR estimates should be interpreted cautiously. A two-tailed *p*-value < 0.05 was considered statistically significant. Statistical analyses were performed using SPSS version 25.0 (IBM Corp., Armonk, NY, USA).

## 3. Results

A total of 29 preterm neonates were included in the analysis. The mean gestational age was 32.0 ± 2.6 weeks, with a mean birth weight of 1748 ± 545 g and a mean length of 41.7 ± 5.0 cm. Thirteen infants (44.8%) were female, and sixteen (55.2%) were male. All neonates were delivered by cesarean section, and delayed cord clamping was performed in 76% (22/29) of cases. The median Apgar score at 1 min was 7 [6,7,8], while the median Apgar score at 5 min was 8 [8,9]. Maternal morbidity was present in 62% (18/29) of cases, with preeclampsia being the most frequent condition (38%).

Respiratory morbidity was common in this cohort. Respiratory distress syndrome (RDS) was observed in 48% of neonates, followed by transient tachypnea of the newborn (10%) and pulmonary adaptation syndrome (7%). Mechanical ventilatory support was required in 20.7% (6/29) of neonates.

LONS occurred in 31% (9/29) of neonates. Among microbiologically confirmed LONS cases, the most frequently identified pathogens were *Klebsiella pneumoniae* and *Escherichia coli*, followed by *Staphylococcus epidermidis*. Detailed timing of LONS onset beyond 72 h of life was not systematically recorded and therefore could not be further analyzed. PCR testing was not routinely performed in all neonates with suspected sepsis during the study period and was requested selectively according to clinical judgment and test availability. No PCR-positive/culture-negative cases were identified in the final cohort. Among the 9 cases of LONS, 5 were confirmed by positive blood culture, while the remaining 4 fulfilled clinical criteria for sepsis without microbiological confirmation.

Nosocomial pneumonia was identified in 10% (3/29) of neonates and was analyzed as a separate outcome; therefore, it was not included among LONS cases. No statistically significant differences were observed between neonates with and without LONS regarding gestational age (31.08 vs. 32.98 weeks, *p* = 0.071), birth weight (1637 vs. 1798 g, *p* = 0.498), sex (*p* = 0.130), Apgar score at 5 min (*p* = 0.375), delayed cord clamping (*p* = 1.00), maternal morbidity (*p* = 0.694), respiratory morbidity (*p* = 0.149), or vitamin D status (*p* = 0.910). However, neonates with LONS had significantly lower Apgar scores at 1 min compared with those without LONS (6 vs. 7, *p* = 0.028).

The mean serum 25-hydroxyvitamin D level at birth was 35.5 ± 13 ng/mL (range: 12.3–65 ng/mL). Vitamin D status was classified as sufficient in 62% of neonates, insufficient in 28%, and deficient in 10%. Mean vitamin D levels were similar between neonates with LONS (35.0 ± 12.0 ng/mL) and those without LONS (35.7 ± 13.7 ng/mL), with no statistically significant difference (*p* = 0.899). Likewise, vitamin D deficiency was not associated with an increased risk of LONS. A weak, non-significant correlation was observed between serum 25-hydroxyvitamin D levels and gestational age (Spearman’s r = 0.32, *p* = 0.090).

Vitamin D deficiency was observed in 3 neonates (10.3%). The unadjusted association between vitamin D deficiency and LONS showed an OR of 1.13 (95% CI 0.09–14.28; *p* = 1.00), calculated from a 2 × 2 contingency table. However, the wide confidence interval reflects substantial statistical imprecision due to the limited sample size and the low number of deficient neonates. Due to the limited number of neonates with vitamin D deficiency, additional subgroup analyses were not performed. Information regarding postnatal vitamin D supplementation was not systematically recorded and therefore could not be analyzed (Table 1).

## 4. Discussion

In this retrospective observational study, serum 25-hydroxyvitamin D levels at birth were predominantly within the sufficient range among preterm neonates. Although lower vitamin D levels were hypothesized to be associated with an increased risk of late-onset neonatal sepsis (LONS), no statistically significant differences were observed between neonates with and without LONS. While no statistically significant differences were identified between groups, clinically meaningful imbalances cannot be excluded due to the limited sample size and exploratory nature of the study. The incidence of LONS observed in our cohort was comparable to rates previously reported in high-risk NICU populations [29].

Our findings are consistent with those reported by Say et al. [29], who also found no significant association between vitamin D levels and LONS in preterm neonates. However, previous studies evaluating the relationship between neonatal vitamin D status and sepsis have shown conflicting results [34,35,36]. These inconsistencies may be explained by important differences in study design and patient populations. Some studies included exclusively preterm neonates, whereas others evaluated predominantly term or mixed neonatal populations. Because preterm neonates have distinct immune immaturity, nutritional reserves, and postnatal adaptation, direct comparison across these groups may be limited.

Differences in the timing and biological source used for vitamin D assessment may also contribute to the variability of published findings. Some investigations measured maternal serum vitamin D during pregnancy, and others analyzed umbilical cord blood at delivery, while several studies, including ours, evaluated early neonatal serum samples obtained shortly after birth. These approaches are not necessarily equivalent, as cord blood and maternal serum concentrations may not fully reflect the dynamic physiological changes occurring during the immediate neonatal period [33]. In addition, postnatal adaptation, fluid shifts, and early nutritional interventions may influence neonatal vitamin D concentrations during the first hours of life.

Another relevant factor is the prevalence of vitamin D deficiency across populations. In our cohort, only 10% of neonates met criteria for vitamin D deficiency, and most infants had vitamin D levels within the sufficient range. This relatively low prevalence may have limited the ability to detect significant associations. In contrast, studies reporting significant associations between vitamin D deficiency and neonatal sepsis have frequently been conducted in populations with substantially higher rates of maternal and neonatal deficiency [31,37]. Geographic location, maternal supplementation practices, socioeconomic conditions, nutritional status, and environmental sun exposure may all contribute to these population-specific differences [32].

Variability in sepsis definitions may further explain discrepancies among studies. Some investigations evaluated early-onset sepsis (EOS), whereas others focused on LONS. These conditions differ substantially in pathophysiology, microbiological profile, and timing of exposure. EOS is more strongly associated with maternal and perinatal factors, whereas LONS is frequently related to nosocomial exposure, invasive procedures, prolonged hospitalization, and postnatal immune adaptation. Consequently, the contribution of vitamin D to infectious susceptibility may differ between EOS and LONS populations. Furthermore, studies differ regarding the inclusion of clinically diagnosed versus exclusively culture-confirmed sepsis cases. Because neonatal blood cultures may have limited sensitivity due to low blood volumes and prior antibiotic exposure, some authors have incorporated clinically suspected or PCR-positive cases into sepsis definitions. These methodological differences may substantially affect outcome classification and comparability across studies.

Previous studies have also associated vitamin D deficiency with respiratory morbidity and prolonged respiratory support [37]. In our cohort, however, no significant association was observed between vitamin D levels and respiratory outcomes. Mechanical ventilation likely reflected greater clinical severity and may have independently contributed to infectious risk through prolonged invasive support and increased exposure to intensive care interventions.

The lower 1-min Apgar scores and higher frequency of mechanical ventilation observed in the LONS group may additionally suggest baseline differences in neonatal clinical severity and vulnerability between groups. Although these differences were not consistently statistically significant, clinically meaningful imbalances cannot be excluded given the limited sample size and exploratory nature of the study. Neonates requiring greater respiratory support or presenting with lower Apgar scores may inherently have a higher susceptibility to infectious complications independent of vitamin D status.

Furthermore, important variables associated with neonatal infectious risk, including central venous catheter exposure, duration of parenteral nutrition, duration of mechanical ventilation, prior antibiotic exposure, and length of hospital stay, were not systematically available in the retrospective records. Consequently, the present study could not adequately adjust for severity-related confounding factors or determine an independent association between vitamin D levels at birth and the subsequent development of LONS. These findings should therefore be interpreted cautiously.

Several limitations should be acknowledged. The relatively small sample size may have reduced statistical power and limited the ability to detect subtle associations. Selection bias cannot be excluded because inclusion required early blood sampling, potentially underrepresenting clinically unstable neonates. The exclusion of early deaths may also have introduced survivor bias. Outcome misclassification remains possible due to the inclusion of clinically diagnosed sepsis together with microbiologically confirmed cases. Residual confounding cannot be ruled out, as potentially relevant variables such as central venous catheter use, duration of mechanical ventilation, parenteral nutrition, invasive procedures, and antibiotic exposure were not systematically assessed. Additionally, although all neonates followed the institutional nutritional protocol recommending vitamin D supplementation after achievement of full enteral feeding, detailed longitudinal information regarding supplementation timing, cumulative dose, adherence, and serial vitamin D monitoring was not consistently available, which may have influenced neonatal vitamin D status and clinical outcomes. Finally, the single-center design conducted in a tertiary NICU may limit the generalizability of these findings.

Although vitamin D has recognized immunomodulatory properties, susceptibility to LONS in preterm neonates is likely multifactorial and influenced by immune immaturity, invasive procedures, prolonged hospitalization, microbiome alterations, and nutritional status. Therefore, isolated vitamin D status at birth may not fully account for infectious susceptibility in this population.

Despite these limitations, neonatal studies remain important for generating preliminary evidence and guiding future hypothesis-driven research. Strengths of this study include the standardized assessment of serum 25(OH)D levels at birth, the focus on a clinically relevant neonatal outcome such as LONS, and the evaluation of a preterm population from a region where evidence remains limited. Importantly, these findings provide preliminary evidence from a Mexican preterm cohort and underscore the importance of considering regional, nutritional, and methodological factors when evaluating the relationship between vitamin D status and neonatal infectious outcomes.

## 5. Conclusions

In this exploratory cohort of preterm neonates, a clear association between serum vitamin D levels at birth and late-onset neonatal sepsis could not be demonstrated. These findings should be interpreted cautiously, given the limited sample size, low prevalence of vitamin D deficiency, and potential residual confounding related to neonatal clinical severity. Larger multicenter prospective studies with longitudinal assessment of maternal and neonatal vitamin D status are needed to better clarify this relationship.

## Figures and Tables

**Table 1:** Comparison of perinatal, clinical, and vitamin D-related characteristics according to LONS status.

Variable	With LONS (*n* = 9)	Without LONS (*n* = 20)	Total (*n* = 29)	*p* Value
Gestational age (weeks), mean ± SD	31.1 ± 2.4	33.0 ± 2.5	32.0 ± 2.6	0.071
Birth weight (g), mean ± SD	1637 ± 510	1798 ± 560	1748 ± 545	0.498
Sex, *n* (%)				0.130 *
• Female	2 (22%)	11 (55%)	13 (45%)	
• Male	7 (78%)	9 (45%)	16 (55%)	
Apgar score at 1 min, median [IQR]	6 (5–7)	7 (6–8)	7 (6–8)	0.028
Apgar score at 5 min, median [IQR]	8 (7–8)	8 (8–9)	8 (8–9)	0.375
Umbilical cord clamping, *n* (%)				1.00 *
• Early	2 (22%)	5 (25%)	7 (24%)	
• Late	7 (78%)	15 (75%)	22 (76%)	
Maternal morbidity, *n* (%)				0.694 *
• Yes	5 (56%)	13 (65%)	18 (62%)	
• No	4 (44%)	7 (35%)	11 (38%)	
Mechanical ventilation, *n* (%)	3 (33.3%)	3 (15.0%)	6 (20.7%)	0.26 *
Respiratory morbidity, *n* (%)				0.149 *
• None	1 (11%)	9 (45%)	10 (34%)	
• RDS	7 (78%)	7 (35%)	14 (48%)	
• PAS	0 (0%)	2 (10%)	2 (7%)	
• Transient tachypnea	1 (11%)	2 (10%)	3 (10%)	
Vitamin D status, *n* (%)				0.910 *
• Deficient (<20 ng/mL)	1 (11%)	2 (10%)	3 (10%)	
• Insufficient (20–29 ng/mL)	2 (22%)	6 (30%)	8 (28%)	
• Sufficient (≥30 ng/mL)	6 (67%)	12 (60%)	18 (62%)	

Abbreviations: LONS, late-onset neonatal sepsis; SD, standard deviation; IQR, interquartile range; RDS, respiratory distress syndrome; PAS, pulmonary adaptation syndrome. * Fisher’s exact test.

## Data Availability

The data presented in this study are available from the corresponding author upon reasonable request. The data are not publicly available due to ethical and privacy restrictions involving patient confidentiality and institutional regulations regarding the use of clinical data.

## Abbreviations

The following abbreviations are used in this manuscript:

NS	Neonatal sepsis (NS)
EOS	Early-onset sepsis
LONS	Late-onset neonatal sepsis
NICU	Neonatal intensive care unit
